# Cooling field and temperature dependent exchange bias in spin glass/ferromagnet bilayers

**DOI:** 10.1038/srep13640

**Published:** 2015-09-08

**Authors:** W. B. Rui, Y. Hu, A. Du, B. You, M. W. Xiao, W. Zhang, S. M. Zhou, J. Du

**Affiliations:** 1 National Laboratory of Solid State Microstructures and Department of Physics, Nanjing University, Nanjing 210093, P. R. China; 2 College of Sciences, Northeastern University, Shenyang, 110819, P. R. China; 3 Department of Physics, Tongji University, Shanghai, 200092, P. R. China; 4 Collaborative Innovation Center of Advanced Microstructures, Nanjing, 210093, P. R. China; 5 MOE Key Laboratory for Anisotropy and Texture of Materials, Northeastern University, Shenyang, 110819, P. R. China

## Abstract

We report on the experimental and theoretical studies of cooling field (*H*_FC_) and temperature (*T*) dependent exchange bias (EB) in Fe_*x*_Au_1 − *x*_/Fe_19_Ni_81_ spin glass (SG)/ferromagnet (FM) bilayers. When *x* varies from 8% to 14% in the Fe_*x*_Au_1 − *x*_ SG alloys, with increasing *T*, a sign-changeable exchange bias field (*H*_E_) together with a unimodal distribution of coercivity (*H*_C_) are observed. Significantly, increasing in the magnitude of *H*_FC_ reduces (increases) the value of *H*_E_ in the negative (positive) region, resulting in the entire *H*_E_ ∼ *T* curve to move leftwards and upwards. In the meanwhile, *H*_FC_ variation has weak effects on *H*_C_. By Monte Carlo simulation using a SG/FM vector model, we are able to reproduce such *H*_E_ dependences on *T* and *H*_FC_ for the SG/FM system. Thus this work reveals that the SG/FM bilayer system containing intimately coupled interface, instead of a single SG layer, is responsible for the novel EB properties.

Recently, spin glasses (SG) have attracted much attention and stimulated intensive studies because the combination of quenched spin spatial randomness and frustration create a complex free energy landscape with multiple local energy minima and finding the stable spin states formidable challenging^1^,^2^,^3^. In spite of many theoretical attempts of developing renormalization group^1^,^4^, mean-field approximation^5^ and Monte Carlo simulation^3^,^6^,^7^,^8^ based analyses, the low-temperature (*T*) spin phases as well as the out-of-equilibrium aging dynamics of such materials remain matters of strong debates. Not surprisingly, the complex nature of SG spin structures gives rise to the unique and diverse macroscopic properties in many fields of science. Exchange bias (EB) for ultrahigh-density magnetic recording is one of such areas^9^.

The EB effect in coupled ferromagnet (FM)/antiferromagnet (AFM) system has been studied extensively because of its critical role in spintronic devices and intriguing spin physics. The EB phenomenon manifests itself as a shifted and broadened magnetization-applied field (*M-H*) hysteresis loop along the *H* axis when FM/AFM systems are cooled below an EB blocking temperature (*T*_B_) under an external magnetic cooling field (*H*_FC_)^10^,^11^. Therefore, EB is both *H*_FC_- and *T*-dependent normally. Most of the EB studies show that large enough *H*_FC_ can either saturate a negative EB field (*H*_E_) for FM typed interfacial couplings (*J*_IF_ > 0)^12^,^13^ or induce a positive *H*_E_ for AFM typed *J*_IF_ (< 0)^14^,^15^,^16^. On the other hand, the thermal energy at high *T* will weaken *J*_IF_, and thus *H*_E_ often decreases monotonically with increasing *T* and finally vanishes at *T*_B_. Under certain circumstances, a sign inversion of *H*_E_, i.e., a small positive EB, can be observed in a narrow *T* region just below *T*_B_ for some EB systems^17^,^18^. These abnormal phenomena are argued to be a result of the unidirectional coercivity (*H*_C_) enhancement along the *H*_FC_ direction. Observation of this effect requires a modest *H*_FC_ and it has been only reported in quite few AFM based materials since the origin of positive EB is very different from systems with negative *J*_IF_^14^. Therefore, the study of *H*_FC_ together with *T* dependences of EB is of critical importance for the understanding of EB mechanism.

As mentioned previously, when SG is involved, the EB dependences on *H*_FC_ and *T* can be different from conventional FM/AFM systems. Nayak *et al.*^19^ found that zero-field-cooled (ZFC, *H*_FC_ = 0) *H*_E_ in Heusler compound Mn_2_PtGa is comparable to the field-cooled (FC) value, due to the coexistence of field-induced irreversible magnetic behavior and a SG-like phase. Sabyasachi *et al.*^20^ found that even a strong *H*_FC_ of 80 kOe cannot saturate the *H*_C_ of nanocrystalline La_1/3_Sr_2/3_FeO_3-*δ*_ at 5 K and this behavior was ascribed to the randomness of glassy magnetic phase. Especially, by using a canonical SG alloy (CuMn), Ali *et al.*^21^ first reported the sign-changeable behavior of *T*-dependent *H*_E_ in SG/FM bilayer systems. Subsequently, Yuan *et al.*^22^ and Ali *et al.*^23^ observed the similar *H*_E_ ∼ *T* trend in other SG materials (FeAu and FeCr). Without performing any *H*_FC_-dependent studies, they interpreted those abnormal *T*-dependent EB behaviors either in the framework of the Ruderman-Kittel-Kasuya-Yosida (RKKY) interaction theory^21^,^22^ or by considering the existence of a *T*-driven SG-to-AFM phase transition^23^. However, due to the diverse and entangled spin interactions existed in SG and the fact these interactions can be affected by the field cooling process, the *T*-dependent sign-changeable *H*_E_ should be greatly influenced by *H*_FC_ in the SG/FM bilayers. Theoretically, Usadel and Nowak^24^ have attempted to reproduce the EB phenomena in the SG/FM bilayer systems by applying an Ising model considering a short-range Gaussian distribution of interactions to simulate the SG material. While they were able to predict the decrease in the magnitude of *H*_E_ at low *T* when increasing *H*_FC_, the model failed to obtain the *H*_E_ ∼ *T* behaviors as seen in Ref. 21, 22, 23. To the best of our knowledge, the *experimental* observations of the *H*_FC_ dependence of EB in SG/FM bilayer systems has remained unreported yet, and there are no *unified* non-phenomenological models that can interpret both the *T* and *H*_FC_ dependences of EB in SG based systems.

In this paper, the effects of *H*_FC_ and *T* on EB in the FeAu/NiFe SG/FM bilayers were first investigated experimentally. Phenomena of *H*_E_ sign inversion at the temperatures just below *T*_B_, the decrease (increase) in *H*_E_ magnitude with increasing *H*_FC_ at low *T* (high *T*), the unimodal distribution of *H*_C_ against *T*, and the *H*_FC_-independent *H*_C_ have all been observed. Then using Monte Carlo technique, we exploited a short-range SG vector model, with a combination of disorder and frustration as well as thermodynamic relaxation and successfully reproduced and interpreted the EB phenomena governed by *H*_FC_ and *T*. Distinct from the AFM based systems, this study suggests that the EB effect and especially the sign-changeable (positive EB) behavior in the narrow *T* region just below *T*_B_ in the SG/FM bilayer systems may be inherent, and exists in all exchange biased SG/FM bilayers.

## Results

**Figure 1:**
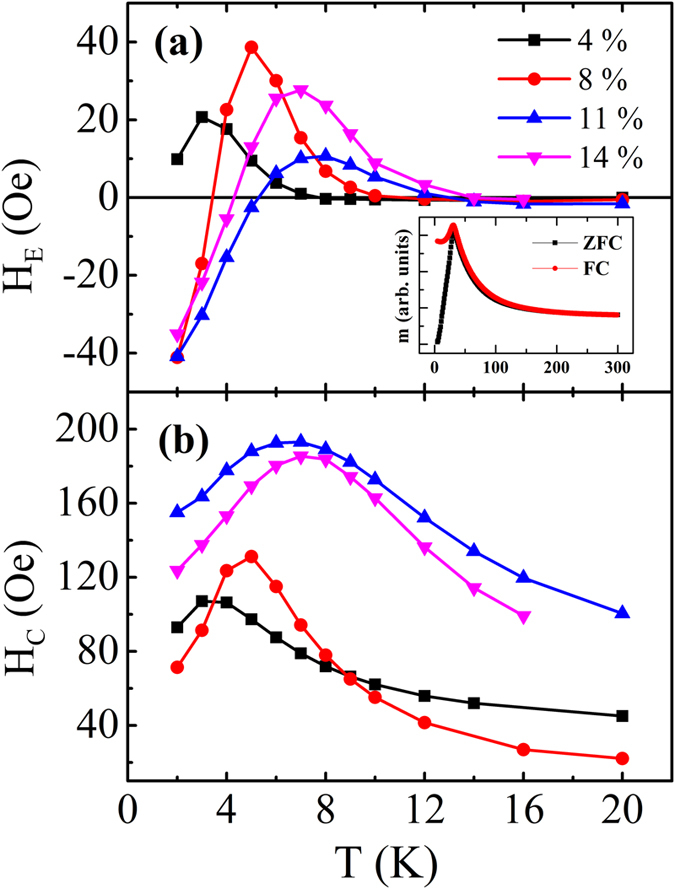
Temperature dependent *H*_E_ (**a**) and *H*_C_ (**b**) with a cooling field of *H*_FC_ = 5 kOe for Fe_*x*_Au_1 − *x*_(50 nm)/FeNi(5 nm) bilayers with *x* = 4%, 8%, 11% and 14%. The inset shows the ZFC-FC magnetization curves for the Fe_11_Au_89_(50 nm) film under *H*_FC_ = 50 Oe with *T* varying between 5 K to 300 K.

The quantities of *H*_E_ and *H*_C_ are calculated based on *H*_E_ = (*H*_C1_* + H*_C2_)/2 and *H*_C_ = (−*H*_C1_* + H*_C2_)/2, where *H*_C1_ and *H*_C2_ denote the coercive fields at the descending and the ascending branches of the *M-H* hysteresis loop, respectively. The temperature dependences of *H*_E_ and *H*_C_ in Fe_*x*_Au_1 − *x*_/FeNi bilayers are shown in Fig. 1, where *x* = 4%, 8%, 11% and 14% and *H*_FC_ is 5 kOe for these measurements. In order to confirm the SG nature of the FeAu layers, ZFC-FC curves were measured with an applied field of 50 Oe. The inset in Fig. 1 shows typical features of spin glass behaviors for a FeAu single layer with *x* = 11%. The value of the freezing temperature (*T*_F_) for this sample is about 30 K, below which the ZFC and FC curves become bifurcated. DC memory effect^25^ has also been observed in this sample (not shown), providing a further proof of the SG state. Similar SG behaviors can be found in all other FuAu single layer films with *x* varying from 4% to 14%.

Figure 1(a) shows a clear sign-change in *H*_E_ versus *T* in the Fe_*x*_Au_1 − *x*_/FeNi bilayers with *x* = 8%, 11% and 14%. With increasing *T* from the lowest value of 2 K, *H*_E_ increases abruptly from a negative value and changes its sign at *T*_0_, which is named the compensation temperature^21^,^22^,^23^. When *T* is increased further, *H*_E_ increases to a positive maximum at another temperature defined as *T*_P_. After that, *H*_E_ decreases gradually and falls below to zero when *T* approaches to *T*_B_. As for the *H*_C_ dependence on *T*, it increases initially with *T* and also shows a concurrent peak at around *T*_P_, as shown in Fig. 1(b). It is noteworthy that *H*_E_ shows a maximum positive value of about 40 Oe when *x* = 8%, much larger in magnitude than any other previous reported results (normally less than 10 Oe), even for a thin FM layer of 2.2 nm in ref. 21. However, the sign-changeable behavior of *H*_E_ with *T* is absent when *x* = 4%, possibly because the value of *T*_0_ is lower than 2 K and beyond the present temperature measuring range.

**Figure 2:**
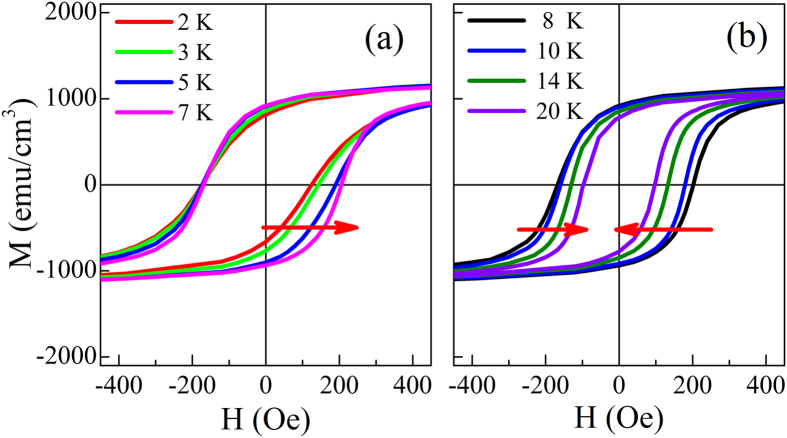
The *M*-*H* hysteresis loops measured for a Fe_11_Au_89_(50 nm)/FeNi(5 nm) sample in the temperature range of 2 K–7 K (**a**) and 8 K–20 K (**b**). The arrows are guides to eyes, indicating the direction of loop shift.

Figure 1 unambiguously demonstrates that for the SG/FM bilayers the *H*_E_ ∼ *T* curve depends strongly on the composition of the SG layer. Previously, such sign-changeable behavior of *H*_E_ against *T* was explained by the competition between long-range oscillatory RKKY couplings from the spins deep inside the SG layer and those close to the interface^21^,^22^. In the present work, besides reconsidering the sign-changeable behavior, we focus on studying the *H*_E_ and *H*_C_ influenced by *H*_FC_. First we discuss the evolution of *M*-*H* hysteresis loops versus *T*. Figure 2 shows the *M*-*H* hysteresis loops obtained between 2 K and 20 K for the Ta(4 nm)/Fe_11_Au_89_(50 nm)/FeNi(5 nm)/Ta(2 nm) sample, after cooled from 300 K to 2 K under *H*_FC_ = 50 kOe. The *M*-*H* hysteresis loop measured at 2 K clearly shows that a negative EB was established after field cooling. As shown in Fig. 2(a), when *T* increases from 2 K to 7 K, the ascending branch of the hysteresis loop shifts rightwards while the descending branch keeps almost unchanged. With further increasing in *T* from 8 K to 20 K, as displayed in Fig. 2(b), the hysteresis loop shrinks from both sides towards the center but the shift of ascending branch is more significantly than the descending branch. Therefore, the different *T*-dependent variation behaviors of the two loop branches lead to the concurrent peak on both the *H*_E_ ∼ *T* and *H*_C_ ∼ *T* curves, coinciding with the appearance of *T*_P_ ∼ 7 K as shown in Fig. 1. These results indicate that the *T*-dependent magnetization reversal mechanisms and/or the thermodynamic spin relaxation at the descending and ascending branches may be quite different.

**Figure 3:**
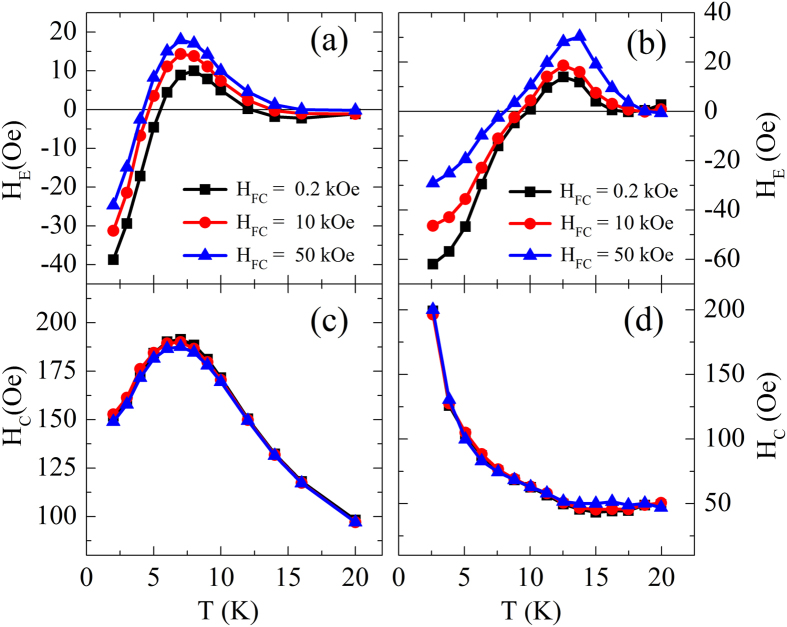
Cooling field influence on the temperature dependent *H*_E_ (**a**) and *H*_C_ (**c**) for a Fe_11_Au_89_(50 nm)/FeNi(5 nm) sample measured between *T* = 2 K and 20 K and the corresponding calculated results of *H*_E_ (**b**) and *H*_C_ (**d**) for the SG/FM bilayers obtained between *T* = 2.6 K and 19.96 K.

Experimental study of the *H*_FC_ dependent EB has been performed and representative results are presented in Fig. 3. As displayed in Fig. 3(a,c), when *H*_FC_ increases from 0.2 kOe to 50 kOe, the *H*_E_ ∼ *T* curve shifts toward upper-left while the *H*_C_ ∼ *T* curves keep almost overlapped. Accordingly, *T*_0_ decreases from 5.5 K to 4.2 K. The peak position of the *H*_E_ ∼ *T* curve moves towards lower *T* slightly and the maximum positive value of *H*_E_ increases significantly from 10 Oe to 18 Oe. Figure 3(b,d) show the corresponding simulation results. The sign inversion in *H*_E_ with *T* can be reproduced and *H*_E_ ∼ *T* curve shift can also be repeated. It is noticed that simulated *T*_0_, *T*_P_, and *H*_E_(*T*_P_) results have certain deviation from the experiments, but the theoretical *H*_E_ ∼ *T* curves agree well with experimental measurements. On the other hand, the calculated trend of *H*_C_ versus *T* is different from the experimental results obtained both by Ali *et al.*^21^ and us, but in agreement with those reported in ref. 22. Another disagreement with the present experimental results is that the theoretical *H*_C_ ∼ *T* curves are *H*_FC_-dependent at the temperatures just below *T*_B_, i.e., *H*_C_ for larger *H*_FC_ is slightly larger. These discrepancies will be addressed later.

**Figure 4:**
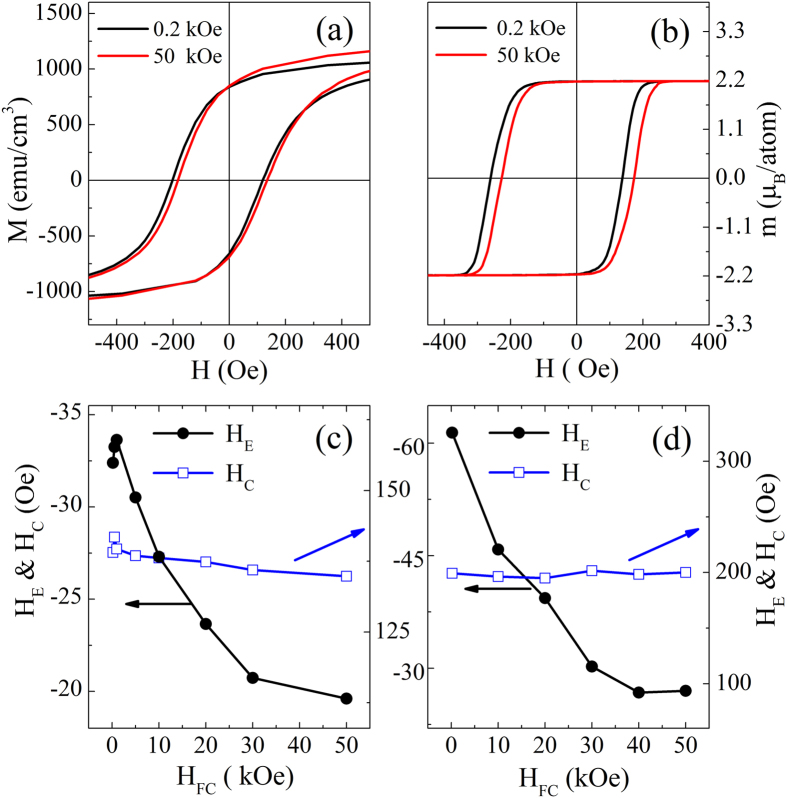
(**a**) The *M*-*H* hysteresis loops measured under *H*_FC_ = 0.2 kOe and 50 kOe at *T* = 2 K for a Fe_11_Au_89_(50 nm)/FeNi(5 nm) sample, (**b**) the calculated *M*-*H* hysteresis loops under *H*_FC_ = 0.2 kOe and 50 kOe at *T* = 2.6 K for the SG/FM bilayers, and the experimental (**c**) and calculated (**d**) cooling field dependences of low-temperature *H*_E_ and *H*_C_.

At low *T*, the *H*_FC_ dependence of *H*_E_ is also interesting. The experimental and simulation results as shown in Fig. 4 indicate that the entire *M-H* hysteresis loop moves rightwards with a strong enough *H*_FC_. As a result, *H*_E_ decreases with increasing *H*_FC_ while *H*_C_ is insensitive to *H*_FC_. Furthermore, there is a slight vertical magnetization shift observed experimentally under strong *H*_FC_, arising from a minor magnetization in the FeAu SG. Increase in *H*_E_ at low *H*_FC_ (≤1 kOe) is very small because saturation in the FeNi (FM) has not been achived.

**Figure 5:**
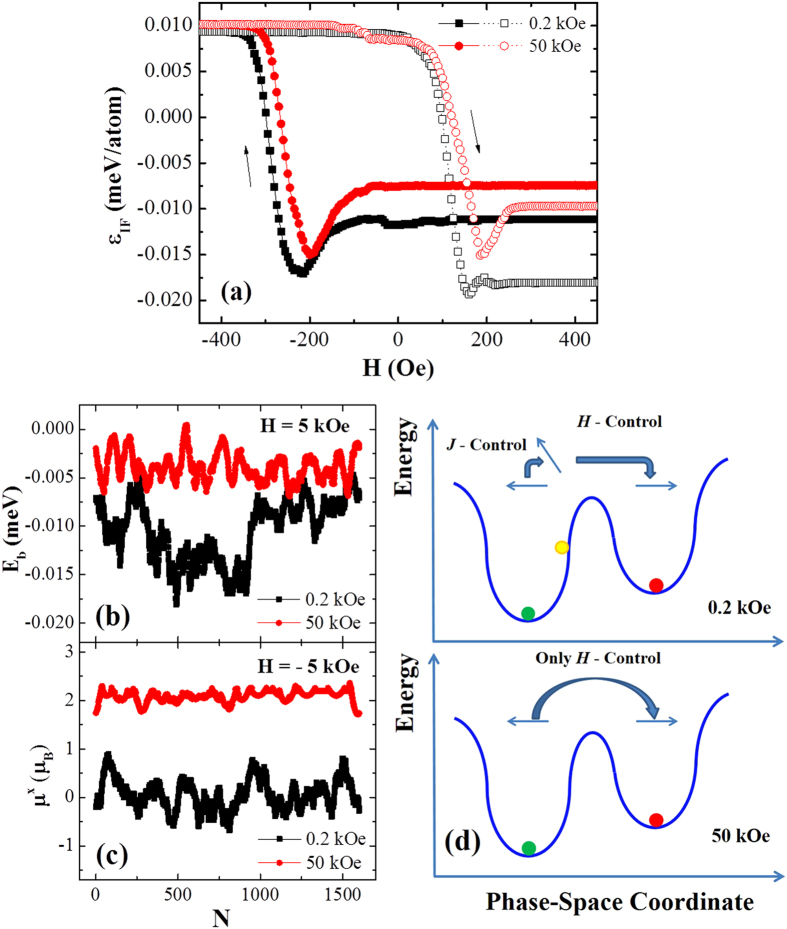
(**a**) Calculated interfacial exchange energy density (*ε*_IF_) during the magnetizing process at *T* = 2.6 K after cooling under *H*_FC_ = 0.2 kOe and 50 kOe, where solid symbols-solid lines and open symbols-dot lines correspond to the descending and ascending branches of the *M-H* hysteresis loops and arrows indicate the magnetizing directions. (**b**) Calculated energy barriers (*E*_b_) of the SG spins at the interface at *T* = 2.6 K after the field cooling process and before the isothermally magnetizing. (**c**) Calculated *x* component (*μ*^*x*^) of the SG magnetic moments at the interface under *H* = −5 kOe during the magnetizing process at *T* = 2.6 K. (**d**) Schematic illustrations of the energy versus phase-space coordinate during the magnetization reversal at the ascending branch of the *M-H* hysteresis loops.

Based on the consistent *H*_E_ results between experiment and simulation, we interpret the above experimental phenomena relying on our simulation method. At first, we have excluded that the EB phenomena in the SG/FM bilayers depend solely on interfaces [see Supplementary Material A]. In other words, the configurations/spins inside the SG influence the FM spins through the SG/FM interface and the SG/FM interface itself also plays a significant role in establishing EB. Thus in the low *T* region, we calculate the interfacial exchange energy density (*ε*_IF_) to interpret the relative EB behavior, which can be expressed as
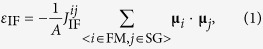
where 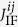 is the interfacial coupling stength between the FM and SG spins, *A* is the interface area and **μ** denotes the magnetic moment of the interfacial spin belonging to the FM or SG. Since this is the dominant energy term influencing the *H*_E_ in SG/FM bilayers and meanwhile a low enough *T* will suppress thermal fluctuation, the change of *ε*_IF_ mainly determines the evolution of spin configuration at the interface during isothermally magnetizing at low *T*. Therefore, it provides us the opportunity to image the *M-H* behaviors microscopically and helps us to elucidate how *H*_E_ is influcenced by *H*_FC_. Figure 5(a) shows the results calculated for *T* = 2.6 K. In addition, other parameters including the spin energy barrier and the *x* component of spin under specific fields are calculated simultaneously in order to provide a clear physical picture during isothermally magnetizing at low *T*, with the results shown in Fig. 5 (b,c), respectively.

At low *T* after field cooling, as shown in Fig. 5(a), *ε*_IF_ for *H*_FC_ = 50 kOe is higher than that for *H*_FC_ = 0.2 kOe, and the value of *ε*_IF_ keeps constant with decreasing *H* in the positive direction. Then *H* changes its sign and increases in magnitude, and the value of *ε*_IF_ begins to decrease around *H* = −100 Oe. For *H*_FC_ = 0.2 kOe, *ε*_IF_ can reduce to a lower value. With further increase in *H* along the negative direction, *ε*_IF_ begins to increase, corresponding to the magnetization reversal at the descending branch, and then reaches a comparable stable value both for *H*_FC_ = 0.2 kOe and 50 kOe. After the reversal, *ε*_IF_ is unchanged approximately again so long as *H* is applied in the negative direction. Once *H* turns back to the positive direction and increases exceeding 100 Oe, *ε*_IF_ decreases rapidly for both *H*_FC_ accompanied by magnetization reversal at the ascending branch. Moreover, the rapid decrease of *ε*_IF_ occurs at a larger *H* for *H*_FC_ = 50 kOe.

The above hysteretic behavior of calculated *ε*_IF_ and the *H*_FC_ dependent EB at low *T* (*T* < *T*_0_) can be understood as follows. After field cooling, the bilayers undergone different *H*_FC_ possess different *ε*_IF_, as a result of the competition between *H*_FC_ and *J*_IF_. Meantime, the competition also establishes a *H*_FC_ dependent energy barrier (*E*_b_) configuration, as shown in Fig. 5(b). These energy barriers confine spin rotation, and *E*_b_ for *H*_FC_ = 50 kOe is apparently higher than that for *H*_FC_ = 0.2 kOe. The reason is that *H*_FC_ is applied along with the SG anisotropy and high *H*_FC_ drags more spins to align with it, so the anisotropy energy of these spins is minimized simultaneously. This will result in a higher *E*_b_ according to the Stoner-Wohlfarth theory. When *H* decreases along the direction of *H*_FC_, no extra energy is required to change the configurations of bilayers, and the systems are called in frozen states. When *H* reverses its direction to become opposite to *H*_FC_ (termed negative direction), there is a critical field (*H *≈ −100 Oe) beyond which the spins in the FM single layer can be reversed^24^. However, in the SG/FM bilayers the SG spins, via *J*_IF_, can impede this process, while the interfacial spins still tend to rearrange towards low exchange energy direction. The rearrangement of SG spin is also influenced by *E*_b_ and thus *ε*_IF_ for *H*_FC_ = 0.2 kOe decreases to a lower value. It is worth noting that the lower the *ε*_IF_ is, the tighter is the bond between spins. To reverse the FM spins to the negative direction, larger *H* is needed for *H*_FC_ = 0.2 kOe to overcome the exchange energy. Hence the magnitude of *H*_C1_ for *H*_FC_ = 0.2 kOe is larger than that for *H*_FC_ = 50 kOe.

When the magnetization reversal at the descending branch is accomplished, *ε*_IF_ is increased abruptly up to a nearly identical value for both *H*_FC_ and the bilayer spin configuration become frozen again. We have to resort to other approaches to interpret the subsequent magnetizing process. Accordingly, the SG spin orientations at the interface (denoted by *μ*^*x*^) under large negative *H* are calculated and shown in Fig. 5(c), and μ^x^ = 2.2 μB indicates that the spins are aligning with *H*_FC_ while *μ*^*x*^ = 0 means that the spins are perpendicular to *H*_FC_. Remarkably, the SG spins for *H*_FC_ = 0.2 kOe show random orientations in the film plane, whereas for *H*_FC_ = 50 kOe all spins keep aligning with *H*_FC_, although in both cases *ε*_IF_ has almost the same value. The spin configuration difference under different *H*_FC_ will influence the magnetization reversal at the ascending branch around *H* = 100 Oe. For *H*_FC_ = 0.2 kOe, randomly oriented SG spins are more easily to drag the FM spins to deviate from the negative field direction via *J*_IF_ under weak *H*, minimizing the interfacial exchange energy. It resembles that *J*_IF_ favors the reduction in the reversal field of FM spin, as illustrated in the top panel of Fig. 5(d). In comparison, for *H*_FC_ = 50 kOe, collinearly aligned SG spins couple to the FM spins via positive or negative *J*_IF_. For the FM spins pointing to the negative field direction, the interfacial coupling with negative *J*_IF_ restrains the FM reversal while positive *J*_IF_ favors the FM reversal. However, the FM reversal for positive *J*_IF_ must overcome the ansotropy energy. Therefore, in both cases *J*_IF_ demotes FM reversal, and thus the FM reversal is driven only by *H* [see the bottom panel of Fig. 5(d)]. As a result, *H*_C2_ for *H*_FC_ = 0.2 kOe is smaller than that for *H*_FC_ = 50 kOe and *H*_E_ for *H*_FC_ = 0.2 kOe is more pronounced.

On the other hand, the influence of *H*_FC_ on *H*_C_ at low *T* will be briefly discussed here. It is well-known that *H*_C_ is determined by the density of pinned sites, such as defects, which controls the nucleation and propagation of domain walls in FM^26^,^27^. In the present SG/FM bilayers, *H*_C_ also depends on the SG spin behaviors at the interface^28^. As mentioned above, *H*_FC_ can reduce |*H*_C1_| while enhance *H*_C2_. We speculate that the decrement of |*H*_C1_| and the increment of *H*_C2_ are comparable due to invariable *E*_b_ during isothermally magnetizing. Therefore, *H*_C_ becomes *H*_FC_-independent.

**Figure 6:**
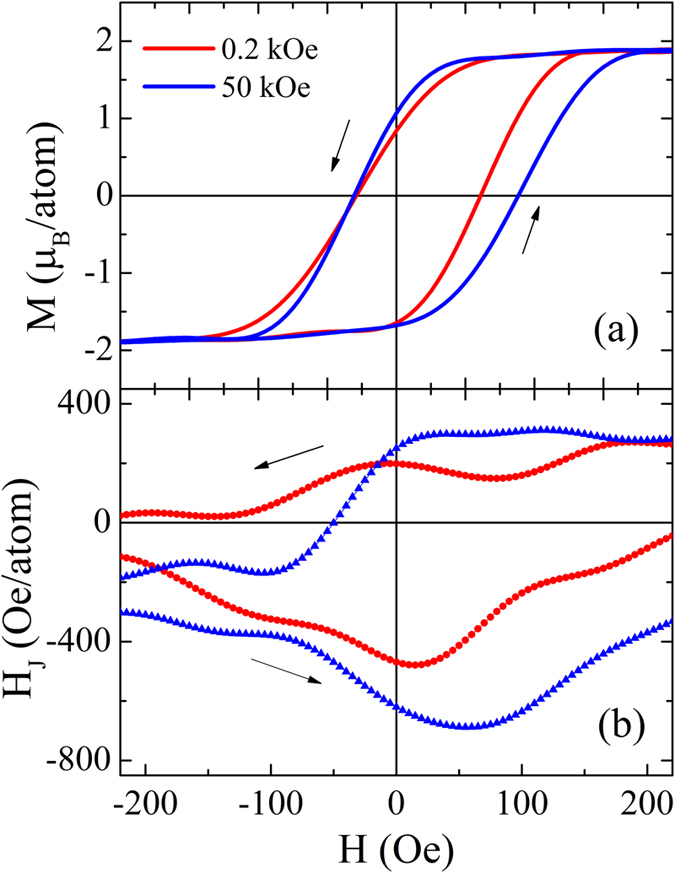
(**a**) Calculated *M-H* hysteresis loops at *T* = 12.52 K after cooling under *H*_FC_ = 0.2 kOe and 50 kOe, and (**b**) the corresponding calculated interfacial exchange fields (*H*_J_) as a function of magnetic field, where arrows indicate the magnetizing directions.

When the *M*-*H* hysteresis loops are measured at the temperatures between *T*_0_ and *T*_B_ (*T*_0_ < *T* < *T*_B_), *H*_E_ may change its sign to become positive and its magnitude is also *H*_FC_-dependent. However, in contrast to *T* < *T*_0_, now *H*_E_ increases with increasing *H*_FC_, as the results calculated at *T* = 12.52 K shown in Fig. 6(a). The *M-H* hysteresis loop for *H*_FC_ = 50 kOe has a larger width and shifts more towards right than that for *H*_FC_ = 0.2 kOe. For the phenomenon of positive *H*_E_ in this *T* region, some mechanisms have been proposed. For example, it was attributed to the AFM interfacial coupling^14^,^15^,^16^ or unidirectional coercive field enhancement along the *H*_FC_ direction in AFM/FM bilayers. It was also argued that the positive *H*_E_ in SG/FM bilayers is directly due to the RKKY type interaction influenced by *T* to a different extent^21^,^22^ or the *T*-driven SG-to-AFM phase transition^23^. However, all these interpretations lack microscopic evidence and do not take into account the influence of *H*_FC_ on positive *H*_E_.

Different from the case of low *T*, thermal fluctuations are enhanced at these temperatures, although the SG spins can be still frozen below *T*_F_. Especially for the FM spins with weak anisotropy, their orientations will be randomized by thermal energy, so *ε*_IF_ cannot well describe the behaviors of SG spins and is no longer suitable to interpret the phenomena at these temperatures. Therefore, we turn to calculate the average interfacial exchange field (*H*_J_), which arises from the SG and interacts with the FM spins through the interface as
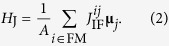
Here 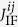 is the interfacial coupling between the FM and SG spins, *A* is the interface area and **μ**_*j*_ represents the magnetic moment of the SG spins at the interface that are directly coupled to the FM spin. Apparently, *H*_J_ is influenced by the SG and thus is *T*- and *H*-dependent. As shown in Fig. 6(a,b), *H*_J_ remains positive until *H*_C1_ and then changes to negative before *H*_C2_. Similar to the external magnetic field effect, *H*_J_ favors the spins to align with it and thus impedes the *H*-driven magnetization reversals for both branches. For *H*_FC_ = 0.2 kOe or 50 kOe, |*H*_J_| in the fourth quadrant is generally larger than that in the second quadrant with same applied magnetic field, i.e. |*H*_J_ (−*H*_C1_)| > |*H*_J_ (*H*_C1_)|. *H*ence a larger *H* is needed to realize magnetization reversal at the ascending branch, i.e., *H*_C2_ is larger than |*H*_C1_|. It results in positive *H*_E_. For larger *H*_FC_ of 50 kOe, *H*_J_ before *H*_C1_ is close to that for *H*_FC_ = 0.2 kOe, leading to similar *H*_C1_ for both *H*_FC_. However, in the fourth quadrant, |*H*_J_| for *H*_FC_ = 50 kOe is much larger than that for *H*_FC_ = 0.2 kOe [see Fig. 6(b)], leading to a larger *H*_C2_ [see Fig. 6(a)]. Consequently, the positive *H*_E_ is larger for higher *H*_FC_.

## Discussion

Different from conventional AFM/FM bilayers, the spin configuration can be rearranged in the present FeAu/FeNi (SG/FM) bilayers even at very low *T* (e.g. 2 K) due to intrinsic randomness and frustration in the SG layer. Significantly, our work reveals that this reconfiguration can be controlled by *H*_FC_ and behaves quite differently in different *T* regions. When *T* < *T*_0_, the spin reconfiguration in the SG can easily occur. For small *H*_FC_, the polarization of SG is weak. In the SG/FM bilayers, a saturated FM layer will couple to the SG spins unidirectionally and result in prominent asymmetry of FM reversal at both branches of an *M*-*H* loop and a negative *H*_E_. However, for large *H*_FC_, the polarization of SG is enhanced, impeding the reconfiguration process. Thus the asymmetry of FM reversal is weakened and *H*_E_ decreases. The experimental and theoretical findings are both consistent with the explanations proposed by Usadel and Nowak^24^. On the other hand, when *T*_0_ < *T* < *T*_B_, the thermal fluctuation cannot be ignored and even may smear out other energy landscapes. The *ε*_IF_ adopted at the low *T* below *T*_0_ is not suitable for the explanation of positive EB effect. Therefore, more direct effect from SG to FM, i.e. *H*_J_ is considered. Positive (negative) *H*_J_ favors the FM spins to align with (against) *H*_FC_ and thus forms FM (AFM) type interfacial coupling to the FM spins along the *H*_FC_ direction. As the calculated results of *H*_J_ shown in Fig. 6(b), an FM-to-AFM transition type of interfacial couplings does occur and this process is *H*-driven, which is different from the *T*-driven phase transition model suggested by Ali *et al.*^23^. Hence a positive *H*_E_ appears when *T*_0_ < *T* < *T*_B_. Moreover, although the thermal fluctuation deteriorates thermal stability, large *H*_FC_ leads to strong polarization of SG and enhance the system stability, which is beneficial to *H*_J_ and favors enhancement in *H*_E_ [see Fig. 6]. As a result, the positive EB for large *H*_FC_ is more obvious than that for small *H*_FC_.

Now we will discuss some of discrepancies between experiment and simulation briefly. In the actual FeAu/FeNi bilayers, when *T* is decreased, the exchange and anisotropy energies are enhanced gradually to induce *H*_E_ below *T*_B_ and this process also contributes to *H*_C_ enhancement. With further decreasing *T*, due to the polycrystalline nature of the FM layer, the easy axes of the FM grains cannot fully lie along with the magnetizing direction, leading to reduction of *H*_C_. Therefore, the variation of *H*_C_ versus *T* is non-monotonic. In the present simulation, a single crystal model including a uniaxial anisotropy and a saturated FM was adopted to study the EB properties and the magnetizing directions were set to be along with the easy axis. Therefore, the calculated *M-H* hysteresis loops are more rectangle-shaped and the calculated values of *H*_E_ or *H*_C_ are larger than those obtained from experiments. Significantly, the increase of anisotropy energy at low *T* in the simulation further contributes to the increase of *H*_C_ when the *M-H* hysteresis loops are measured along the easy-axis direction. As a result, *H*_C_ has a monotonic and sharp increase with decreasing *T*. Also, due to the saturated FM in the initial state, the experimental result of increase in *H*_E_ with initially increasing *H*_FC_ is not obtained in simulation. Since the model only considers the most dominant energy terms concerning EB, a perfect agreement between theory and experiment cannot be achieved.

To summarize, the dependence of EB properties in FeAu/FeNi SG/FM bilayers on *H*_FC_ and *T* have been studied experimentally and numerically. A sign-changeable behavior of *H*_E_ versus *T* is observed, accompanied by a nonmonotonic behavior of *H*_C_ versus *T*. More significantly, a phenomenon of decrease (increase) in magnitude of *H*_E_ at low (high) *T* with increasing *H*_FC_ is first observed in experiment while *H*_C_ is always insensitive to *H*_FC_ below *T*_B_. It is also found that the mechanisms of negative and positive *H*_E_ influenced by *H*_FC_ are quite different. Therefore, the highlight of this paper includes exhibiting the *H*_FC_ modulation of the *T*-dependent EB behaviors in SG/FM bilayers experimentally and interpreting these behaviors simultaneously by using a unified vector model.

## Methods

### Sample fabrication and measurement

The samples were deposited on silicon wafer by DC magnetron sputtering at room temperature with a stacking sequence of Ta(4 nm)/Fe_*x*_Au_1 − *x*_(50 nm)/FeNi(5 nm)/Ta(2 nm). Here, *x* denotes the atomic fraction of Iron in FeAu alloy. The FeAu layer was co-sputtered with tilted iron and gold guns and the sample composition was characterized by Energy Dispersive X-Ray Spectroscopy (EDX). By fixing the sputtering power of iron and varying that of Au, *x* was varied from 4% to 14%. A 4 nm Ta buffer layer was deposited to promote the SG/FM morphology and a 2 nm Ta capping layer was deposited to prevent sample from oxidation. FeNi represents Fe_19_Ni_81_, which is used as the magnetic pinned layer. The base pressure for sputtering was better than 7.0 × 10^−6^ Pa and the working Ar pressure was kept at 0.3 Pa during film deposition. Another series of Fe_*x*_Au_1 − *x*_ single layer films with the identical composition and thickness (i.e., 50 nm) were also deposited on Kapton substrates as reference samples for the low-*T* SG characterization by means of the ZFC and FC *M*-*T* measurements.

Magnetic hysteresis loop measurements were performed in a SQUID-VSM (Quantum Design) with the applied magnetic field (in the range from −5 kOe to 5 kOe) parallel to the film plane. Before each *M*-*H* hysteresis loop measurement at a specific *T*, a magnet reset procedure (oscillatory demagnetization between ±1 Tesla) was performed in order to train the sample to an approximately equilibrium state to eliminate the EB training effect and reducing the residual magnetic field to less than 3 Oe. This will ensure that the obtained *H*_*E*_ and *H*_*C*_ are accurate with an error less than 3 Oe.

### Theoretical model and calculation details

On atomic scale, the real coarse-grained SG/FM bilayers are simulated by a finite number of spins placed on the nodes of a simple cubic lattice. By means of effective size scaling, a lateral dimension of 40 × 40 units in the film plane and a film thickness of 5 monolayers are used with periodic boundary conditions only applying in the plane. Simulations were performed with the Heisenberg model, with the Hamiltonian written as
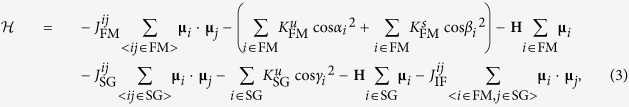
where **μ**_*i*_ denotes the magnetic moment of the spin at site *i*. The first term describes the direct exchange energy in the FM. The next two terms are the magnetocrystalline and shape anisotropy energies of the FM, where *α*_*i*_ (*β*_*i*_) is the angle between **μ**_*i*_ in the FM and the *x* (*z*) axis. The fourth term is the Zeeman energy of the FM, where **H** is applied along the *x* axis. From the fifth to seventh term represent the direct exchange, magnetocrystalline anisotropy, and Zeeman energies of the SG. Here *γ*_*i*_ is the angle between **μ**_*i*_ in the SG and the *x* axis. And the last term is the interfacial direct exchange energy between FM and SG. Detailed scaling and parameterizing processes have been described in the Supplementary Material B.

The simulation protocol mimics the experimental process. Initially, a cooling process under an *H*_FC_ is performed on the SG/FM bilayers, where the FM spins are all pointing to the *H*_FC_ direction and the SG spins are randomly oriented, from *T* = 300 K to a target temperature. Then, an isothermal magnetization is recorded by cycling *H* from 5 kOe to −5 kOe to extract *H*_E_ and *H*_C_. As for the update of spin state, a *path-related* Metropolis algorithm^29^ is adopted during the Monte Carlo simulation, which has been also introduced in the Supplementary Material B in details. This calculation is repeated for 10^4^ times per spin to find the equilibrium state of the system, and then an additional 10^5^ steps are taken to measure the thermodynamic average of the magnetization. Finally, 200 independent realizations of the disorder are averaged to reduce the statistical errors.

## Additional Information

**How to cite this article**: Rui, W. B. *et al.* Cooling field and temperature dependent exchange bias in spin glass/ferromagnet bilayers. *Sci. Rep.* **5**, 13640; doi: 10.1038/srep13640 (2015).

## Supplementary Material

Supplementary Information
