# Supplementary material for: Cooling field and temperature dependent exchange bias in spin glass/ferromagnet bilayers
Source: Sci Rep. 2015 Sep 8;5:13640. doi: 10.1038/srep13640 (PMC4562234; doi:10.1038/srep13640)
Supplement: Supplementary Information [file srep13640-s1.pdf]

Cooling field and temperature dependent exchange bias in spin glass/ferromagnet bilayers

W. B. Rui[1], Y. Hu[2, 5, *], A. Du[2], B. You[1, 4], M. W. Xiao[1], W. Zhang[1], S. M. Zhou[3], J. Du[1, 4, *]

[1]*National Laboratory of Solid State Microstructures and Department of Physics, Nanjing University, Nanjing 210093, P. R. China*

[2]*College of Sciences, Northeastern University, Shenyang 110819, P. R. China*

[3]*Department of Physics, Tongji University, Shanghai 200092, P. R. China*

[4]*Collaborative Innovation Center of Advanced Microstructures, Nanjing 210093, P. R. China*

[5]*MOE Key Laboratory for Anisotropy and Texture of Materials, Northeastern University, Shenyang 110819, P. R. China*

*Corresponding author: huyong@mail.neu.edu.cn, jdu@nju.edu.cn

SUPPLEMENTARY MATERIAL

A. Comparison of hysteresis loops between SG/FM and AFM/FM bilayers

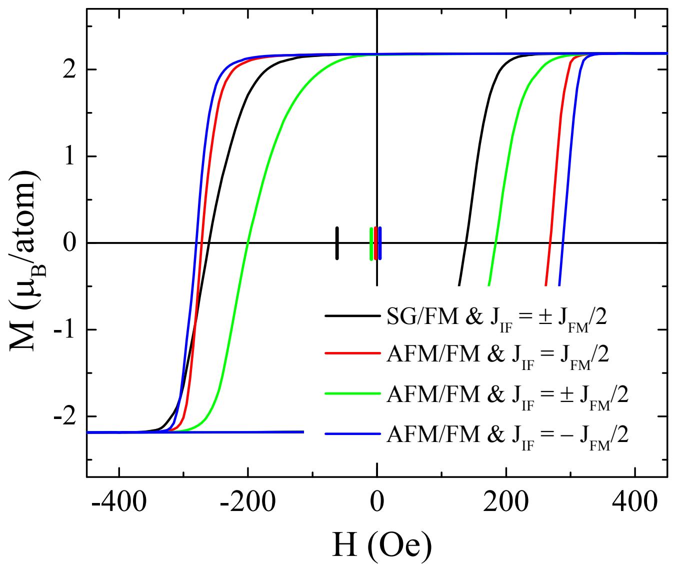

Fig. S1 Calculated *M-H* hysteresis loops in the SG/FM and AFM/FM bilayers with different types of J_{IF} at T = 2.6 K after cooling under H_{FC} = 0.2 kOe, where short lines are positioned at the center of loops.

In order to prove that the abnormal EB phenomena observed in our SG/FM bilayers are not solely originated from the interface, which is always regarded as the source of EB in most of heterostructures, a model of AFM/FM bilayers is also established with AFM exchange coupling constant $J_{AF} = -J_{FM}/2$ and different types of J_{IF} for comparison. The calculated *M-H* hysteresis loops at T = 2.6 K after cooling under H_{FC} = 0.2 kOe are exhibited in Fig. S1. It is generally acceptable that the EB effect is weak in the AFM based systems after cooling under a negligible H_{FC} (close to ZFC), regardless of the type of J_{IF}. This is due to that the AFM is nearly compensated after the above field cooling process, which cannot generate obvious EB. On the contrary, a pronounced EB in the SG based systems after cooling under the same weak H_{FC} is observed, which implies that the origin of EB in the SG based systems is quite distinct from that in the AFM based systems. It also proves that the EB effect in SG/FM bilayers is dependent on the SG bulk. Malozemoff [1-3] and Mauri *et al* [4] presented a random-field model in their pioneering works and discussed the interfacial domain formation to consider the contamination, pinholes, or roughness at the FM/AFM interface, and finally,

improved the theoretical results down to the right order. Our calculated results do not violate their theories, because the type and strength of J_{IF} can also change the EB behaviors when H_{FC} is increased (Results are not shown). In one word, both the SG bulk and SG/FM interface are responsible for the EB behaviors in SG/FM bilayers.

B. Details of model establishment and algorithm modification

For the bilayers with dimensions in the range of those experimentally investigated, it is far from being a straightforward calculation due to the computational effort beyond present-day standard computational facilities, and thereby quantitative correspondences are difficult generally. On the contrary, a qualitative description is possible if an available scaling approach [5, 6] is used. We suppose the lattice constant of $a_0 \approx 0.227$ nm, and thus only 40×40×5 spins positioned on the simple cubic lattices are enough to simulate the dimensions of 100×100×12.5 nm^3. Periodic boundary conditions are used in the two lateral dimensions (i.e., in the film plane) to eliminate finite-size effects, whereas open boundaries are used for the top and bottom surfaces. Only one-layer FM and four-layer SG are considered to meet EB requirements. Occasional checks are also performed with larger lateral sizes (e.g. 200×200×5) to confirm that our results do not depend on the size of simulated system. With the dimension scaling ratio of 40/(100/0.227) ≈ 0.09, the direct exchange interactions (J) are reduced by a factor of $\sqrt[0.55]{0.09} \approx 0.013$ simultaneously while the anisotropies (K) are kept constant. Referring to the orders of J and K in real materials [7, 8], we set $|\boldsymbol{\mu}_i|$ = 2.2 μ_B (Fe), J_{FM} calculated by $10\,\text{meV}\big/\left(|\boldsymbol{\mu}_i|\right)^2$ times 0.013 (realistic exchange energy = 10 meV), $J_{SG} = J_{IF} = \pm J_{FM}/2$ to guarantee a (nearly) saturated FM below T_F, $K_{FM}^u = 6.5\times10^{-4}\,\text{meV}$ with the easy axis along one cube edge (i.e., along the x axis), $K_{FM}^s = -K_{FM}^u$ to replace the classical dipolar interactions and to confine the rotation of spins in the film plane, and $K_{SG}^u = 1.3\,\text{meV}$. Finally, the unit of H in the simulation is calculated by $H^{sim} = |\boldsymbol{\mu}_i|J_{FM} \approx 1$ Tesla, so it has the same dimension as that in the experiment, i.e., $H^{sim} = H^{exp}$. But the unit of T in the simulation has to be set through comparing the simulated T_B to the experimental one obtained from the sample of $Fe_{11}Au_{89}$ (50 nm)/FeNi (5 nm).

Another significant issue is to simulate the spin configuration of the FeAu SG,

because in the simplest mean-field approximation the SG ordering is nonunique and the thermodynamic states are degenerate, i.e., a macroscopic property may correspond to different microscopic configurations. The details of these states will depend on the precise values of interactions and so vary from sample to sample. Moreover, the RKKY interactions exist in the FeAu SG and may play a key role in the novel EB behaviors like in the CuMn SG [9]. Nevertheless, if the alloy is very dilute, positive and negative signs of J will be equally likely and the decay of the J strength as the minus third power of distance is rapid. A large number of randomly chosen J are uniformly distributed throughout the SG and an average over them is zero. In line with the proposal of Zhan *et al* [10], we simplify these long-range interactions to describe the SG by the short-range $\pm J$ frustration model [11, 12] so that the quantities of J_{SG} and J_{IF} are independent random variables setting the values $\pm J_0$ ($J_0 > 0$) according to the probability distribution

$$P\left[J^{ij}\right] = p\delta\left(J^{ij} - J_0\right) + \left(1-p\right)\delta\left(J^{ij} + J_0\right), \tag{1}$$

where $p = 0.5$ denotes the concentration of positive bonds (i.e., FM interactions) being randomly distributed in the systems. They are unchanged during the simulations (quenched disorder).

Finally, a modified Monte Carlo technique is proposed to update the spin states. In the standard Monte Carlo Metropolis algorithm [13], changes of the spin orientation are accepted or rejected according to a single-site transition probability,

$$W = \min\{1, \exp[-\left(E_{trial} - E_{initial}\right)/k_B T]\}. \tag{2}$$

Remarkably, the probability is T-dependent and only determined by the energy difference between trial (E_{trial}) and initial states ($E_{initial}$) of the spin. However, the spin energy is a function of polar and azimuthal angles of the spin. That is, the spin energy is instantly changing during the spin rotation, which should be also responsible for W. Accordingly, Du *et al* [14] has proposed a *path-related* Metropolis algorithm. In a time unit (i.e., in a Monte Carlo step), they calculate the curved surface of energy of every spin to judge whether energy maxima exist in the rotation path from the initial to trial state. If so, E_{trial} in equation (2) is replaced by the saddle-point energy (E_{saddle}), which is defined as the minimum value of the energy maxima, to determine the acceptance or rejection of the trial state. Thus, it reads

$$W = \min\{1, \exp[-\left(E_{saddle} - E_{initial}\right)/k_B T]\}. \tag{3}$$

C. Magnetic hysteresis loop measurements from only the FeAu layer

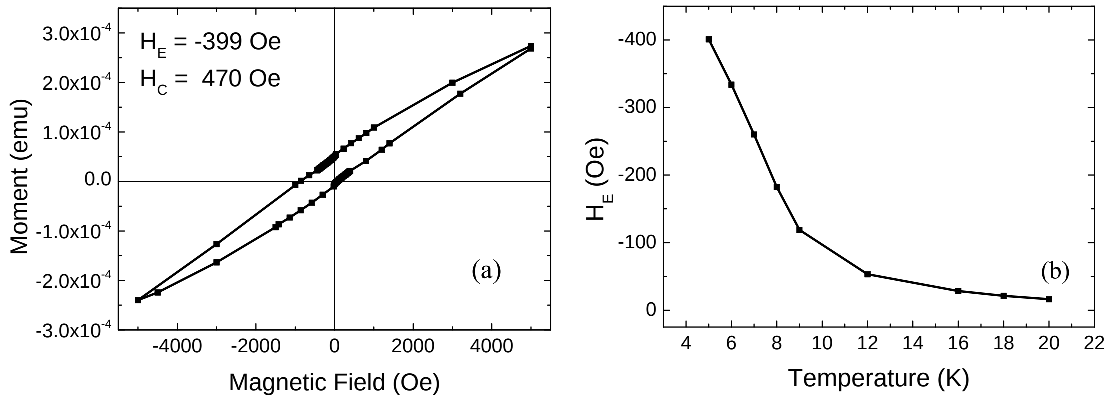

Fig. S2 (a) *M-H* loop measured at T = 5 K and (b) Temperature dependent H_E after field cooling under H_{FC} = 0.5 kOe for the $Fe_{11}Au_{89}$ single layer.

In order to confirm that the EB effect in the present SG/FM system does not *solely* come from the SG single layer, we performed the *M-H* loop measurements from the $Fe_{11}Au_{89}$ SG single layer, with the results displayed in Fig. S2. In order to enhance the recorded magnetic signal, the $Fe_{11}Au_{89}$ layer was deposited with its thickness of 120 nm on a Kapton substrate (tape) with 5 cm long and 0.3 cm wide. The sample was first heated to 300 K and then cooled to 5 K under H_{FC} = 0.5 kOe for later measurements. The *M-H* loop measured at T = 5 K is displayed in Fig. S2(a), which shows obvious shift along the horizontal axis with the calculated H_E and H_C much bigger than those reported in the present FeAu/FeNi bilayer samples. However, in the same measurement range from 5 kOe to -5 kOe as that performed on those FeAu/FeNi bilayer samples, the *M-H* loop for the single FeAu layer is much inclined, suggesting that the loop shift is possibly caused by minor loop effect. More significantly, the quantity of H_E decreases monotonically with increasing T and no sign-changeable behavior can be observed at all from 5 K to 20 K, as clearly shown in Fig. S2(b). Therefore, the novel EB phenomena in the present work are impossible to come from the SG layer solely.

References

[1] Malozemoff, A. P. Random-field model of exchange anisotropy at rough ferromagnetic-antiferromagnetic interfaces. *Phys. Rev. B* **35**, 3679-3682

(1987).

[2] Malozemoff, A. P. Mechanisms of exchange anisotropy (invited). *J. Appl. Phys.* **63**, 3874-3879 (1988).

[3] Malozemoff, A. P. Heisenberg-to-Ising crossover in a random-field model with uniaxial anisotropy. *Phys. Rev. B* **37**, 7673-7679 (1988).

[4] Mauri, D., Siegmann, H. C., Bagus, P. S. & Kay, E. Simple model for thin ferromagnetic films exchange coupled to an antiferromagnetic substrate. *J. Appl. Phys.* **62**, 3047-3049 (1987).

[5] d'Albuquerque e Castro, J., Altbir, D., Retamal, J. C. & Vargas, P. Scaling Approach to the Magnetic Phase Diagram of Nanosized Systems. Phys. Rev. Lett. **88**, 237202 (2002).

[6] Vargas, P., Altbir, D. & d'Albuquerque e Castro, J. Fast Monte Carlo method for magnetic nanoparticles. Phys. Rev. B **73**, 092417 (2006).

[7] Staemmler, V. & Fink, K. An ab initio cluster study of the magnetic properties of the CoO(001) surface. Chem. Phys. **278**, 79-87 (2002).

[8] Schrön, A., Rödl, C. & Bechstedt, F. Crystalline and magnetic anisotropy of the 3d-transition metal monoxides MnO, FeO, CoO, and NiO. Phys. Rev. B **86**, 115134 (2012).

[9] Ali, M. et al. Exchange bias using a spin glass. Nat. Mater. **6**, 70-75 (2007).

[10] Zhan, X., Mao, Z., Xu, X., Chen, X. & Kleemann, W. Spin disorder dependence of the exchange bias effect. Phys. Rev. B **86**, 020407 (2012).

[11] Toulouse, G. Theory of the frustration effect in spin glasses: I. Commun. Phys. **2**, 115 (1977).

[12] Binder, K. & Young, A. P. Spin glasses: Experimental facts, theoretical concepts, and open questions. *Rev. Mod. Phys.* **58**, 801-976 (1986).

[13] Landau, D. P. & Binder, K. *A Guide to Monte Carlo Simulations in Statistical Physics* [Landau, D. P. & Binder, K. (ed.)] [150-152] (Cambridge University Press, Cambridge, 2005).

[14] Du, H. F. & Du, A. The hysteresis curves of nanoparticles obtained by Monte Carlo method based on the Stoner-Wohlfarth model. *J. Appl. Phys.* **99**, 104306 (2006).
